# Towards prevention of new COVID-19 infections in institutions of higher education: factors influencing compliance with mask-wearing among public university students in Ghana

**DOI:** 10.1186/s12879-024-09110-9

**Published:** 2024-02-21

**Authors:** Fred Yao Gbagbo, Richmond Opoku, Rosemary Quarcoo

**Affiliations:** 1 Department of Health Administration and Education, University of Education, Winneba, Ghana; 2 Department of Clothing and Textiles Education, University of Education, Winneba, Ghana

**Keywords:** Compliance, COVID-19, Ghana, Mask-wearing, Students, University

## Abstract

**Background:**

Ghana’s mask-wearing compliance with COVID-19 prevention protocols has not been as impressive among the general population. In this study, we examined factors influencing compliance with mask-wearing among public university students in Ghana to make recommendations for the prevention of new COVID-19 infections in public universities.

**Methods:**

We conducted this Census in a public university in Ghana between January and December 2022. The study design was an exploratory-cross-sectional and online survey. Structured questionnaires developed by the authors were used to collect data from 3,272 students. Data were analyzed with Jeffreys’s Amazing Statistics Program (JASP). Frequency distributions were used to summarize the data into tables and graphs whilst logistic regression analysis was done to examine the factors influencing compliance with mask-wearing among participants as well as their mask-wearing behaviors in school.

**Results:**

Compliance with mask-wearing measures was high with 85.9% of the students wearing a nose mask always or often. Agreeing that the reusable masks do not last long was associated with a reduced chance of non-compliance (OR = 0.70, 95% CI = 0.57, 0.86). However, agreeing with some challenges was associated with increased chances of non-compliance. These included concerns that it is boring to mask after wearing makeup or having a haircut (OR = 1.71, 95% CI = 1.37, 2.14), and that masking is burdensome because it has to be removed when talking (OR = 1.26, 95% CI 1.01, 1.57), and that it is difficult to hear while masked (OR = 1.36, 95% CI = 1.04, 1.79).

**Conclusion:**

Cost-benefit analyses, opinions about one’s look, and communication difficulties are the key factors influencing students’ non-compliance with mask-wearing regulations. To encourage student compliance with mask-wearing regulations at Ghana’s public universities, we recommend innovation in nose mask manufacture.

## Background

The emergence of Severe Acute Respiratory Syndrome Coronavirus 2 (SARS-CoV-2; COVID-19), had compelled most countries to endorsed the use of restrictive protocols such as social distancing and wearing of face/nose masks as means of reducing infections and deaths from the virus [1–3]. These measures do not only reduce an individual’s chance of contracting the virus but also the possibility of infecting others [4, 5]. Scientifically proven measures such as wearing a nose/face mask have been effective particularly since the main mode of spread of the virus is airborne transmission [6–8]. 

In Ghana, nose/face mask wearing were mandatory preventive measures in public places including schools during the COVID-19 Pandemic [2]. although mask-wearing has been proven as one of the most effective ways of limiting the spread of the virus, especially in a situation where COVID-19 vaccination rates are relatively low, [9, 10] compliance with mask-wearing protocols in the general population and among students has not been impressive [2, 11]. A study conducted during the first wave of the pandemic at transport stations in the central business district of Accra found that face masks were either not worn or worn by a small proportion of passengers in over 90% of the stations studied [11]. Also, a recent study among students found that only 31.5% of students wore face masks often or always [2]. This is a very worrying finding given that most public schools in Ghana are often overcrowded as a result of limited infrastructure [12]. 

An issue of public health concern particularly in institutions of higher learning that are strategically situated and may be of immense use for sensitization, health promotion and education on preventing COVID-19, is the factors influencing compliance with mask-wearing among public university students in Ghana; an area which has not been adequately studied. One study explored students’ perceptions about the pandemic and found that students who perceived COVID-19 to be easily transmissible and deadly were more likely to wear nose masks [2]. However, the study [2] only focused on a handful of issues, mainly disease-related perceptions of students (i.e., perceptions about transmissibility and severity of COVID-19 ) but failed to provide a comprehensive assessment of several other issues such as concerns with physical appearance, health problems associated with mask-wearing, challenges with communication, social influences, and subjective cost-benefit evaluations of nose mask-wearing, all of which are likely to influence compliance with mask-wearing protocols [13–15]. 

Since the effectiveness of mask mandates depend on generalized public compliance, [16] public health practitioners need to understand why people in places of high human density including institutions of higher learning may not comply with mask-wearing measures. This understanding is necessary, not only for planning preventive interventions in the current pandemic era but also for planning emergency response measures for future events that may require the use of nose/face masks. Against this background, is this study to chart a path forward by examining the factors influencing compliance with mask-wearing among public university students in Ghana.

## Methods

### Study area

The study was conducted among students in a public university in Ghana. The choice of this particular university was purposive. The main objective of our study was to ‘examine the factors that influence compliance with mask-wearing among public university students in Ghana’. The mandate of the university where data was collected (the study area) is generally to train educators/teachers for schools. We believe that the outcome of teacher training is to impact knowledge acquired to students which invariably impact to change in behavior to a large extent. It is in relation to this that we deem the objectives of our study is well aligned with the mandate of the university selected for our study. In line with the ethical requirements and conditionality for approving the study protocol, the details about the identify of this university is being anonymized to ensure optimal confidentiality and privacy of the data collected and findings.

### Study design, participants, and data collection procedures

The study design was an exploratory-cross-sectional online Census using a structured questionnaire for data collection. Being a university-wide census conducted online, we anticipated all students inclusiveness/participation in the survey because invitation for participation in the study was extended to all students in the university and they were expected to respond to an online Census questionnaire that was available between January and December 2022. The Census designed with Google forms was advertised on various student social media platforms including major departmental WhatsApp and telegram platforms, through various faculty and departmental association leaders. The Census was designed to accept only one response from each respondent. The researchers did not offer any material rewards for participation in the survey. In all, a total of 3,272 valid responses (constituting 10.3% of the targeted population) were received and included in the final analysis of the study.

### Variable measures

#### Outcome variable

The main outcome of interest in this study was mask-wearing as a behavior among the students. Students were asked to indicate how often they wore nose masks in places where they were required to do so (i.e., in public). Four Likert-type response categories were provided (always, often, sometimes, and never). Students who always or often wore nose masks in public places were categorized as compliant, coded as “0” and those who sometimes or never wore nose masks in public places were categorized as non-compliant, coded as “1”.

### Predictor variables

The predictor variables, also known as independent variables, are inputs or factors that were used to predict the value of the outcome /independent variables. In this study, it constitutes the aspect of the data set that was measured to determine its effects on the dependent variable.

In all, a total of 18 items relevant variables were included in the survey. The items were measured as Likert-type items with four categorical levels (strongly disagree, disagree, agree, and strongly agree). Students were deemed to agree if they selected strongly agree or agree on items and were coded as “1” while those who strongly disagree or disagree were categorized as disagree and coded as “0”. The scale demonstrated good internal consistency with a Cronbach’s α of 0.84 after deleting one item resulting in a 17-item scale used for the final data analysis. Details on all the items can be found in the results section of this work.

### Data analysis

Data were analyzed with Jeffreys’s Amazing Statistics Program (JASP) [17]. Frequencies and percentages were used to summarize the data collected on the sociodemographic characteristics of students, compliance with mask-wearing protocols, and agreement with predictor variables. To determine the relative importance of various issues in predicting compliance with mask-wearing protocols, a logistic regression analysis was conducted. The coding of variables was done in such a way that odds ratios (OR) greater than 1 indicated an increased chance of non-compliance if participants agreed with a predictor item. Variables that showed statistically significant effects in the univariate analysis (*p* < 0.05) were fitted into a multivariate model using forward selection.

## Results

### Sociodemographic characteristics of participants

The results (Table 1) showed that most of the students were males (52.8%). Also, the majority of them were within the age range of 21–25 years (68.5%). Most of the responses were obtained from students who studied a program in the social sciences (56.6%) and a large majority of the students were in level 100.

**Table 1:** Sociodemographic Characteristics of Respondents (*N* = 3272)

Variable	Frequency	Percentage
Gender		
Male	1728	52.8
Female	1544	47.2
Age		
18–20 years	402	12.3
21–25 years	2,233	68.5
26–30 years	505	15.5
≥ 31 years	121	3.7
Programme		
Sciences	473	14.9
Business	901	28.5
Social sciences	1,789	56.6
Level of study		
Level 100	1,186	37.4
Level 200	1,075	33.8
Level 300	696	21.9
Level 400	219	6.9

### Compliance with mask-wearing measures among participants

**Fig. 1:**
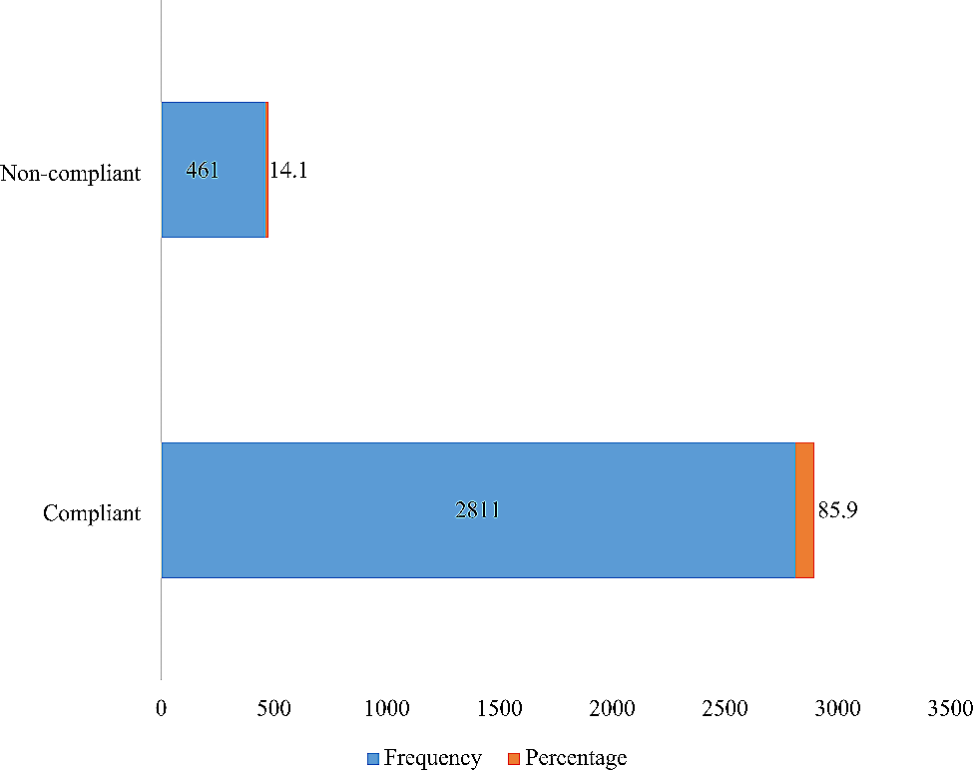
Distribution of students on compliance with nose mask measures

The results of the study showed that compliance with mask-wearing measures was high among the students who participated in the study with 85.9% of them wearing nose masks always or often whenever in public places (Fig. 1). However, 14.1% of the participants were non-compliant with mask-wearing measures despite strict mask-wearing regulations on the university campus.

### Concerns influencing mask-wearing among study participants

The results of the univariate analysis of the concerns influencing mask-wearing have been presented in Table 2. The results showed that the majority of students (i.e., > 50%) agreed with all the concerns on health and safety as well as communication. Two items each under the physical appearance and social influence domains saw the majority of students agreeing with them. However, none of the items under cost-benefit considerations saw the majority of students in agreement with them (i.e., < 50% agreed).

**Table 2:** Univariate analysis of issues predicting mask-wearing behaviors among participants

		Risk of non-compliance	
	% Agree	OR	95% CI
**Health and Safety Concerns**			
I cannot breathe properly through the nose mask	80.4	1.19	0.92, 1.53
Wearing face masks for an extended period causes strain and pain around my ears	73.3	1.01	0.81, 1.26
Wearing face masks for an extended period causes rashes/acne/itch on my nose and/or ears	73.4	0.94	0.75, 1.17
**Cost-Benefit Considerations**			
I cannot afford to buy disposable nose masks regularly	33.0	1.06	0.86, 1.30
The reusable nose masks do not last long	45.0	0.76	0.62, 0.93
Wearing a nose mask does not help prevent the spread of the virus in any way	31.1	1.00	0.81, 1.24
The type of mask I prefer is expensive	38.1	0.98	0.80, 1.20
Wearing a nose mask is not necessary because Covid-19 is not a deadly disease among the youth	16.6	0.80	0.60, 1.06
**Perceptions of Physical Appearance**			
Wearing nose masks hides my beauty	56.0	1.10	0.90, 1.34
Wearing a nose mask does not let me look presentable in public	35.7	1.06	0.87, 1.30
It is boring to put on a nose mask after wearing nice makeup or having a nice haircut	58.4	1.76	1.42, 2.17
**Social Influence**			
Some of my lecturers do not wear the mask so I do not think it is necessary	60.6	1.03	0.84, 1.26
In the community, I live or at home, most people do not wear the mask so I don’t see why campus should be different	37.4	1.06	0.87, 1.30
My friends or roommates do not wear their masks so I do not think it is necessary	55.0	1.09	0.89, 1.33
**Communication Challenges**			
I have to remove the mask anytime I want to talk and I find that burdensome	53.1	1.46	1.19, 1.78
Sometimes I cannot hear colleagues talking when I am masked	72.6	1.82	1.42, 2.34
I find it difficult to talk for my colleagues to hear even when they are close to me	54.0	1.42	1.16, 1.74

The results of the univariate logistic analysis showed that five variables from across three domains significantly predicted mask-wearing. Agreeing that the reusable nose masks do not last long was associated with a reduced chance of non-compliance with mask-wearing measures (OR = 0.76, 95% CI = 0.62, 0.93). Those who agreed with the concern that it is boring to mask after wearing makeup or having a haircut had an increased chance of non-compliance with mask-wearing measures (OR = 1.76, 95% CI = 1.42, 2.17). Also, those who agreed that they had to remove the mask anytime they had to talk which made masking burdensome had an increased chance of not masking (OR = 1.46, 95% CI = 1.19, 1.78). Agreeing that it is difficult to hear while masked was associated with an increased chance of non-compliance with mask-wearing measures (OR = 1.82, 95% CI = 1.42, 2.34). Finally, agreeing that people around them find it difficult to hear what they say when masked was also associated with an increased likelihood of not masking (OR = 1.42, 95% CI = 1.16, 1.74).

In the final multivariate model, four variables showed statistically significant independent influences on the mask-wearing of the students (Table 3). These variables included concerns that the reusable nose masks do not last long (OR = 0.70, 95% CI = 0.57, 0.86), it is boring to mask after having a nice makeup or haircut (OR = 1.71, 95% CI = 1.37, 2.14), having to remove the mask anytime they wanted to talk (OR = 1.26, 95% CI 1.01, 1.57), and finally, difficulty hearing what others say while masked (OR = 1.36, 95% CI = 1.04, 1.79). However, concerns that others sometimes find it difficult to hear what they say were not significantly associated with non-compliance with mask-wearing measures in the multivariate analysis (OR = 1.16, 95% CI = 0.93, 1.45).

**Table 3:** Multivariate analysis of issues predicting mask-wearing behaviours among participants

	Risk of non-compliance	
	OR	95% CI
The nose masks do not last long but are expensive	0.70	0.57, 0.86
It is boring to put on a nose mask after wearing nice makeup or having a nice haircut	1.71	1.37, 2.14
I have to remove the mask anytime I want to talk and I find that burdensome	1.26	1.01, 1.57
Sometimes I cannot hear colleagues talking when I am masked	1.36	1.04, 1.79
I find it difficult to talk for my colleagues to hear even when they are close to me	1.16	0.93, 1.45

## Discussions

The authors examined factors influencing compliance with mask-wearing among students in a public university in Ghana. The study found high compliance to COVID-19 related mask-wearing measures of the university. Recent research from Ethiopia found that 89.5% of university students adhered to mask-wearing measures amid the COVID-19 pandemic [18]. Our findings are in line with the Ethiopian study [18] because research findings from both institutions have indicated that a significant majority of university students appear convinced to wear nose masks as a preventive measure against the COVID-19 pandemic. We observed, however, that 14.1% of our participants were not complying with mask-wearing mandates despite the strict enforcement efforts by university authorities. It is worthy to note that in a country like Ghana, where vaccination rates are not very promising, individual preventive measures such as mask-wearing are an important aspect of the fight against COVID-19 as well as future public health emergencies of similar characteristics as Covid [9, 10]. 

Regarding the concerns that influence mask-wearing among the students, three broad issues of concern influenced the mask-wearing of the students (i.e., cost-benefit considerations, perceptions of physical appearance, and communication challenges). We observed that students who perceived reusable nose masks as less expensive and a cost-effective way to prevent contracting COVID-19 infection were more likely to mask. A possible explanation for this finding might be the documented dislike for non-reusable masks among a majority of university students. A study among university students in Vietnam found that only 23.1% (168/728) of participants preferred to use cloth masks and only 43.5% (73/168) of this number reused their masks [19]. The study also observed that university students had a poor understanding of the procedures for cloth masks re-use [19]. The implication of this is that students in our study who agreed that reusable masks do not last long might prefer surgical masks and can afford them since the levels of agreement to the two concerns on cost were very low. Consequently, they are more likely to put on their affordable surgical masks compared to those who prefer reusable cloth masks but have a poor understanding of how to maintain them.

Concerning issues with physical appearance, students expressing concerns about the inconvenience of masking after having a nice make-up or haircut were less likely to wear a nose mask. This is not a surprising result since young people are more inclined to act in ways that will enhance their chances of having the positive judgment of people around them [20]. This is especially true for female students, given the fact that females are more concerned about their appearance due to deep-rooted social values and perceptions about how women should appear physically [21, 22]. The practical implication of this finding is that interventions aimed at improving compliance with mask mandates among students and particularly among females, who are much conscious of their physical appearance should focus on the production and use of transparent masks and face shields.

The authors also examined concerns about communication and all items under communication were statistically significant in the univariate models while two items were significantly associated with mask-wearing behaviors of the students in the final multivariate model. Students agreeing with the inconveniences associated with communication while masked were less likely to wear nose masks. The consensus in the literature is that facial expressions and gestures play a very significant role in human communication [23]. However, wearing a nose mask conceals facial gestures used in communication, and for people living with impaired hearing or hearing loss, who rely heavily on visual perception of facial expressions and gestures, communication becomes extremely difficult with nose masks [24, 25]. It is therefore imperative for mask producers to be innovative enough by producing masks with windows that allow for some reasonable visual perception of facial expressions while masked.

## Conclusions

In this study, we assessed compliance with COVID-19 -related mask-wearing measures among students in a Ghanaian university and the concerns influencing compliance with mask-wearing directives over a one-year period. The observation that 14.1% of our participants did not adhere to mask-wearing on campus for various reasons despite the strict regulations calls for a reassessment of the policy on nose masking to ensure total compliance. We also recommend innovations in nose mask production and supply to students to enhance full compliance with mask-wearing directives.

### Strengths and limitations

We the authors believe that a significant strength of our study is the methodology used to examine a behavioral issue of public health significance that relates to a pandemic situation. The second strength relates to the interventional outcome towards prevention of new COVID-19 infections in institutions of higher education in Ghana. Additionally, being a census, the findings can be generalized among the entire students’ population of the university for a comprehensive intervention.

Despite the modest strength, the paper has a few limitations. First, we used self-reported data which has the potential of social desirability bias since mask-wearing is a behavior generally approved by society [16, 25]. However, the use of online surveys is known to be one of the most effective means of limiting social desirability bias since it guarantees optimal privacy for participants [26, 27]. Within a social science context, we recommend a qualitative study as a continuation of the present study so as to probe further into the reasons for non-compliance or compliance to masking on campus. Findings from this future qualitative study may provide some valuable information to inform institutional policy and programme decisions.

## Data Availability

The datasets generated and/or analyzed during the current study are available from the corresponding author upon reasonable request.

## Abbreviations

CI: Confidence interval
COVID-19: Corona Virus Disease of 2019
JASP: Jeffreys’s Amazing Statistics Program
OR: Odds Ratio
SARS-CoV-2: Severe Acute Respiratory Syndrome Coronavirus 2

## References

[1] Amoah C, Simpeh F. Implementation challenges of COVID-19 safety measures at construction sites in South Africa. J Facilities Manage (2020).

[2] Apanga P, Awingura (2021). Isaac Bador Kamal Lettor, and Ramatu Akunvane. Practice of COVID-19 preventive measures and its associated factors among students in Ghana. Am J Trop Med Hyg.

[3] World Health Organization. *Advice on the use of masks in the context of COVID-19: interim guidance, 6 April 2020*. No. WHO/2019-nCov/IPC_Masks/2020.3. World Health Organization; 2020.

[4] Kraemer MUG, Yang C-H, Gutierrez B, Wu C-H, Klein B, Pigott DM. The effect of human mobility and control measures on the COVID-19 epidemic in China. Science. 2020;368(6490):493–7. Open COVID-19 Data Working Group†. 10.1126/science.abb4218 PMC7146642 32213647

[5] Leung NHL, Daniel KW, Chu, Eunice YC, Shiu K-H, Chan JJ, McDevitt, Benien JP, Hau H-L, Yen et al. Respiratory virus shedding in exhaled breath and efficacy of face masks. *Nature medicine* 26, no. 5 (2020): 676–680. 10.1038/s41591-020-0843-2 PMC8238571 32371934

[6] Tabatabaeizadeh S-A (2021). Airborne transmission of COVID-19 and the role of face mask to prevent it: a systematic review and meta-analysis. Eur J Med Res.

[7] Zhang R, Li Y, Zhang AL, Wang Y, Mario J. Molina. Identifying airborne transmission as the dominant route for the spread of COVID-19. *Proceedings of the National Academy of Sciences* 117, no. 26 (2020): 14857–14863. 10.1073/pnas.2009637117 PMC7334447 32527856

[8] Haischer MH, Beilfuss R, Hart MR, Opielinski L, Wrucke D, Zirgaitis G, Uhrich TD (2020). Hunter. Who is wearing a mask? Gender-, age-, and location-related differences during the COVID-19 pandemic. PLoS ONE.

[9] Greenhalgh T (2020). Face coverings for the public: laying straw men to rest. J Eval Clin Pract.

[10] Greenhalgh T, Manuel B, Schmid T, Czypionka. Dirk Bassler, and Laurence Gruer. Face masks for the public during the COVID-19 crisis. *Bmj* 369 (2020). 10.1136/bmj.m1435 32273267

[11] Bonful H, Affran A, Addo-Lartey, Justice MK, Aheto JK, Ganle B, Sarfo, Aryeetey R (2020). Limiting spread of COVID-19 in Ghana: compliance audit of selected transportation stations in the Greater Accra region of Ghana. PLoS ONE.

[12] Mensah DK (2019). Teachers’ perspective on implementation of the double track senior high school system in Ghana. Int J Emerg Trends Social Sci.

[13] Howard MC (2021). Gender, face mask perceptions, and face mask wearing: are men being dangerous during the COVID-19 pandemic?. Pers Indiv Differ.

[14] Looi K, Hoe SX, Zhang, Li N. Demographic and Hygienic Factors as Predictors of Face Mask Wearing During COVID-19 Pandemic in Malaysia. *medRxiv* (2021).

[15] Techasatian L, Lebsing S, Uppala R, Thaowandee W, Chaiyarit J, Supakunpinyo C, Panombualert S (2020). The effects of the face mask on the skin underneath: a prospective censusduring the COVID-19 pandemic. J Prim care Community Health.

[16] Galasso V, Pons V, Profeta P, Becher M, Brouard S, Foucault M. Gender differences in COVID-19 attitudes and behavior: Panel evidence from eight countries. *Proceedings of the National Academy of Sciences* 117, no. 44 (2020): 27285–27291. 10.1073/pnas.2012520117 PMC7959517 33060298

[17] van den Bergh D, Wagenmakers E-J. and Frederik Aust. Bayesian Repeated-Measures ANOVA: An Updated Methodology Implemented in JASP. (2022).

[18] Larebo YM, Desta Erkalo A (2021). Knowledge, attitudes, and practices of face mask utilization and associated factors in COVID-19 pandemic among Wachemo University Students, Southern Ethiopia: a cross-sectional study. PLoS ONE.

[19] Duong M, Cuong HT, Nguyen, Bich Thuy Duong (2021). A cross-sectional study of knowledge, attitude, and practice towards face mask use amid the COVID-19 pandemic amongst university students in Vietnam. J Community Health.

[20] Charity Hudley AH, Mallinson C (2017). It’s worth our time: a model of culturally and linguistically supportive professional development for K-12 STEM educators. Cult Sci Edu.

[21] Bidaki R, Majidi N, Ahmadi AM, Bakhshi H, Mohammadi RS, Mostafavi S-A (2018). Mohammad Kazemi Arababadi, Maryam Hadavi, and Afshin Mirzaei. Vitiligo and social acceptance. Clin Cosmet Invest Dermatology.

[22] Zhang Xiu-jie, Wang Ai-ping, Shi Tie-ying, Zhang J, Xu H, Wang Da-qiu, Li Feng (2019). The psychosocial adaptation of patients with skin disease: a scoping review. BMC Public Health.

[23] Mohammadi K. The eyes have it: communication and face masks. Guardian (2020).

[24] Chodosh J, Barbara, East., Weinstein, Blustein J. Face masks can be devastating for people with hearing loss. *bmj* 370 (2020). 10.1136/bmj.m2683 32646862

[25] Jasmine T-C. Coronavirus: Call for clear face masks to be ‘the norm’. *BBC News. Accessed: 30 December* (2021). https://www.bbc.com/news/world-52764355.

[26] Ball HL (2019). Conducting online surveys. J Hum Lactation.

[27] Larson RB (2019). Controlling social desirability bias. Int J Market Res.

